# Cognitive impairment and preferences for current health

**DOI:** 10.1186/1477-7525-7-1

**Published:** 2009-01-09

**Authors:** Joseph T King, Joel Tsevat, Mark S Roberts

**Affiliations:** 1 Section of Neurosurgery, VA Connecticut Healthcare System, West Haven, Connecticut, USA; 2 Department of Neurosurgery, Yale University, New Haven, Connecticut, USA; 3 Section of Outcomes Research, Division of General Internal Medicine, Department of Internal Medicine, University of Cincinnati Medical Center, Cincinnati, Ohio, USA; 4 Center for Clinical Effectiveness, Institute for Health Policy and Health Services Research, University of Cincinnati Medical Center, Cincinnati, Ohio, USA; 5 Veterans Affairs Medical Center, Cincinnati, Ohio, USA; 6 Section of Decision Sciences and Clinical Systems Modeling, Division of General Internal Medicine, Department of Medicine, University of Pittsburgh, Pittsburgh, Pennsylvania, USA; 7 Center for Research on Health Care, University of Pittsburgh, Pittsburgh, Pennsylvania, USA; 8 Division of General Internal Medicine, University of Pittsburgh, Pittsburgh, Pennsylvania, USA

## Abstract

**Background:**

We assessed preferences for current health using the visual analogue scale (VAS), standard gamble (SG), time trade-off (TTO), and willingness to pay (WTP) in patients with cerebral aneurysms, a population vulnerable to cognitive deficits related to aneurysm bleeding or treatment.

**Methods:**

We measured VAS, SG, TTO, and WTP values for current health in 165 outpatients with cerebral aneurysms. We assessed cognitive impairment with the Mini Mental State Examination (MMSE; scores < 24 = cognitive impairment). We examined the distributions of preference responses stratified by cognitive status, and the relationship between preferences and cognitive impairment, patient characteristics, and aneurysm history.

**Results:**

Eleven patients (7%) had MMSE scores < 24. The distribution of preferences responses from patients with cognitive impairment had greater variance (SG, 0.39 vs. 0.21, P = 0.001; TTO, 0.36 vs. 0.24, P = 0.017) and altered morphology (VAS, P = 0.012; SG, P = 0.023) compared to the responses of unimpaired patients. There was good correlation between most preference measures for unimpaired patients (VAS:TTO, rho = 0.19, P = 0.018; SG:TTO, rho = 0.36, P < 0.001; SG:WTP, rho = -0.33, P < 0.001) and a trend towards significance with another pairing (VAS:WTP, rho = 0.16, P = 0.054). In subjects with cognitive impairment, there was a significant correlation only between VAS and TTO scores (rho = 0.76, P = 0.023). Separate regression models showed that cognitive impairment was associated with lower preferences on the VAS (β = -0.12, P = 0.048), SG (β = -0.23, P = 0.002), and TTO (β = -0.17, P = 0.035).

**Conclusion:**

Cognitive impairment is associated with lower preferences for current health in patients with cerebral aneurysms. Cognitively impaired patients have poor inter-preference test correlations and different response distributions compared to unimpaired patients.

## Background

Patient preferences for health states, also known as health values or utilities, are central to decision analysis and cost-effectiveness analysis. There are several methods to assess health state preferences, including the visual analogue scale (VAS), standard gamble (SG), time trade-off (TTO), and willingness to pay (WTP) methods [1-4]. The SG and TTO present the subject with a hypothetical choice involving a risk of immediate death or a shorter life, respectively, in exchange for perfect health, and then calculate preferences based on responses. The VAS, often not considered a true preference measure, asks the subject to rate health states on a linear scale anchored usually by dead and perfect health. WTP offers subjects the option of purchasing a hypothetical treatment producing perfect health, and the purchase price indicates the strength of their preference.

Cerebral aneurysms have a prevalence from 2–6% [5-7], and can adversely affect quality of life via subarachnoid hemorrhage (SAH), mass effect, thromboembolic stroke, psychological distress, and adverse outcomes of surgical or endovascular aneurysm treatment. Up to 50% of patients who experience aneurysmal hemorrhage experience cognitive deficits [8], and deficits can also occur as a complication of elective treatment aimed at preventing aneurysm rupture [9]. Cognitive deficits can affect quality of life. Both the general population and caretakers for patients with Alzheimer's disease report diminished values for dementia health states [10-12], and patients with cognitive impairment have altered response patterns during testing of preferences for current health [13]. As part of a larger study of quality of life in patients with cerebral aneurysms, we examined the effects of cognitive impairment on preferences as measured with the VAS, SG, TTO, and WTP.

## Methods

### Study Population

We enrolled a sample of outpatients with cerebral aneurysms from the University of Pittsburgh Medical Center neurosurgery clinics between June 2001 and February 2004. All neurosurgery clinic patients with a cerebral aneurysm were eligible for inclusion in the study, including patients with a newly diagnosed symptomatic or incidental aneurysm, patients being followed for a known aneurysm, and patients who had recently undergone elective or emergency aneurysm treatment. After obtaining informed consent, the patients underwent a structured interview administered by a research assistant to collect information on demographics, personal habits, comorbid diseases, cognitive functioning, and preferences. Additional data were abstracted from paper and electronic medical records. The protocol was approved by the institutional review boards (IRB) of Yale University and the University of Pittsburgh. Patients received $25 as compensation for completing the interview. Our IRB has determined that payments of this amount are not coercive, and the payments help maximize the participation of the full spectrum of eligible patients.

### Preference Testing

Preferences for the subjects' current state of health were assessed in order with the VAS, SG, TTO, and WTP. The VAS, SG, and TTO were anchored by "perfect health" and "death." Perfect health was defined as "The best possible health that you can imagine. You are cured of your brain aneurysm, and you are cured of all other health problems." Subjects were given a card printed with the anchor point definition as a mnemonic. We used iMPACT3 software [14] for SG and TTO testing, a paper and pencil instrument for the VAS, and a custom Visual Basic program to assess WTP. A research assistant performed preference testing using a script, and recorded when the subject had difficulty understanding or completing one or more of the four preference assessment tasks.

#### Visual Analogue Scale

Subjects were asked to value their current health by placing a mark on a 10 cm line anchored by the words "death" and "perfect health" [1]. Preferences were calculated as the ratio of the distances from death to current health and death to perfect health.

#### Standard Gamble

Subjects were offered a choice between living in their current state of health or accepting a hypothetical treatment for all of their health problems [2]. The treatment had two possible outcomes: "death" or "perfect health." The probabilities of death and cure were varied systematically using a ping-pong technique [15] until the subject was indifferent between their current health and the treatment. The probability of dying was represented graphically on the computer screen by blackening out a corresponding proportion of a grid of 100 faces. The iMPACT3 software permitted probabilities to vary by 1%. The patient's preference score was then calculated as the probability of perfect health at the indifference point.

#### Time Trade-Off

Subjects were offered a choice between continuing in their current state of health or reducing their life span by trading off years of life in exchange for perfect health [3]. The number of years required to obtain perfect health was systematically varied using a ping-pong technique until the subject was indifferent between their current health and the trade-off. We presented all subjects with a 20-year life expectancy, the maximum permitted by the iMPACT3 software; the minimal incremental change permitted by the iMPACT3 software was 1 year, the equivalent of 0.05 utility units. The relationships between 20 years of life in current health, reduced life expectancy in disease-free health, and time lost from early death were displayed by horizontal bars on the computer screen. The patient's preference was calculated as the ratio between time in perfect health and time in current health at the indifference point.

#### Willingness to Pay

We used a closed-ended contingent valuation WTP bidding method to determine WTP for a hypothetical treatment resulting in perfect health [4]. We asked subjects to imagine that they could purchase this treatment with a single payment. Subjects were encouraged to consider the financial consequences of buying the treatment by reading the following statement: "*To pay for your treatment, you might use your savings, your present household income, loans that you would have to pay back, and possible future increases in your income after you have perfect health." *The interviewer then quoted a series of prices to the subject, and for each amount the subject was asked: "*Would you be willing to pay $X for a cure for your health problems?" *A computer program calculated each successive price offer based on an algorithm incorporating annual household income and the subject's last response. Subjects were first asked if they were willing to pay $1. If they were willing to pay $1 (> 98% were), the next price offer was the equivalent value of 1 month's income. Offers were then systematically increased or decreased until convergence on a final monetary value was reached. The maximum WTP value permitted was 10 times the subject's own annual household income.

### Mini-Mental State Examination

After assessments of health values, the interviewer administered the MMSE [16], an 11-item test of cognitive function consisting of 7 tasks designed to measure orientation, memory, attention, and naming, and the ability to follow verbal and written commands, write a sentence spontaneously, and copy a complex polygon. The tasks are scored individually, and scores are summed to yield the standard composite score (range from 0–30). Lower scores represent worse cognitive functioning, and scores < 24 are considered indicative of cognitive impairment. The MMSE has been used to assess cognitive functioning in patients with cerebral aneurysms [9,17-20].

### Data Analysis

Categorical variables were tabulated, and means, standard deviations, and medians were calculated for continuous variables. Characteristics of study patients and excluded patients (i.e., those who did not complete all study instruments) were compared by using Fisher's exact test for categorical variables and the Mann-Whitney U test for continuous variables. The distributions and variances of preferences of unimpaired and cognitively impaired patients were compared using the Kolmogorov-Smirnov test and the folded F test, stratified by preference measurement tool. The correlations between preference measurement tools were measured using Spearman's rho, stratified by cognitive status. Four separate stepwise multivariate linear regression models were developed to explore the relationships between VAS, SG, TTO, and WTP health values versus subjects' characteristics (age, sex, race, education, and income [WTP only]), aneurysm history (previous SAH, prior aneurysm treatment, history of stroke), and cognitive impairment (MMSE < 24). Simple linear regression and a P value < 0.200 were used to select candidate variables for inclusion in the stepwise regression models. Statistical significance was defined by a P value < 0.05; P values ≥ 0.05 but < 0.1 were considered to indicate a trend.

## Results

### Study Population

Two hundred seventeen eligible patients consented to participate in the study, and 165 (76%) completed the VAS, SG, TTO, WTP, and MMSE, comprising the study population. Incomplete data collection was caused by errors in survey completion, research staffing issues (i.e., staff vacation or sick time, simultaneous patients in excess of what available staff could process), and patient time constraints. There was a trend towards excluded patients having a lower rate of stroke (11%) compared to the study patients (22%; P = 0.099). There were no significant differences between the 165 study patients and the 52 excluded patients in terms of age, sex, race, education, income, cognitive impairment, history of SAH, or prior aneurysm treatment (for all, P ≤ 0.110). The mean (SD) patient age was 54.2 (12.5) years; 119 (72%) were women and 151 (92%) were Caucasian (Table 1). Eighty-five patients (52%) had a history of SAH, 112 (68%) had undergone previous aneurysm treatment, and 35 (22%) had a history of stroke.

**Table 1:** Characteristics of the Study Population

		**N = 165**
**Age (years)**	Mean (SD)	51.2 (12.5)
	Range	25 – 90
**Sex**	Female	119 (72%)
**Race**	Non-Hispanic Caucasian	151 (92%)
	Other	14 (8%)
**Education**	High school or technical school graduate	149 (91%)
	Missing	1 (0.6%)
Annual income*	Mean (SD)	$41,100 ($33,800)
	Missing	8 (5%)
**Number of aneurysms**	1	120 (73%)
	2	25 (15%)
	3	15 (9%)
	4	3 (2%)
	5	1 (0.6%)
	7	1 (0.6%)
**Aneurysm locations**	Anterior circulation	210 (87%)
	Posterior circulation	32 (13%)
**Aneurysm status**	All aneurysms obliterated	73 (44%)
	One or more unsecured aneurysms	92 (56%)
**Patients with prior SAH**		85 (52%)
**Patients with prior aneurysm treatments**	Surgical clipping	83 (50%)
	Endovascular embolization	25 (15%)
	Both	4 (3%)
	None	53 (32%)
**History of stroke**		35 (22%)
**MMSE assessment of cognitive functioning**	Mean (SD)	27.5 (2.6)
	Impaired (MMSE 0–23)	11 (7%)
	Unimpaired (MMSE 24–30)	154 (93%)

2003 $US

SAH = subarachnoid hemorrhage

SD = standard deviation

MMSE = Mini Mental State Examination

### Cognitive Impairment

The mean (SD) MMSE score was 27.5 (2.6), and 11 (7%) patients had an MMSE score < 24 consistent with cognitive impairment. There was no association between a history of stroke and cognitive impairment (P = 0.451). Twenty patients (12%) had difficulty understanding or completing one or more preference assessments; however, there was no association between difficulty understanding or completing preference instruments and cognitive impairment (P = 1.000).

### Preferences for Current Health

**Figure 1:**
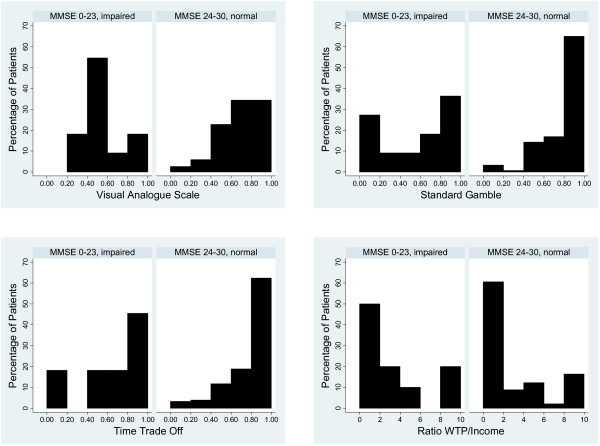
**Cognitive impairment and preferences for current health**. Histograms stratified by cognitive status illustrating preferences for current health measured with the visual analogue scale (VAS), standard gamble (SG), time trade off (TTO), and willingness to pay (WTP). Cognitive impairment is defined as a Mini Mental State Examination (MMSE) score < 24.

The median (intra-quartile range) for each of the preference measures were: VAS: 0.70 (0.52, 0.81), SG: 0.86 (0.70, 0.97), TTO: 0.90 (70, 1.00), and WTP: $35,000 ($6,400, $153,500). A comparison of histograms of each preference measure stratified by cognitive functioning revealed differences in location and distribution of responses (Figure 1). Preferences of patients with normal cognitive functioning had typical skewed-normal (VAS) or skewed (SG, TTO, WTP) distributions with a modal response near perfect health. In contrast, patients with cognitive impairment showed significantly different patterns for VAS (i.e., a quasi-normal distribution with modal values near 0.5; d = 0.461, P = 0.012) and SG (quasi-bimodal distribution with peaks near 0.0 and 1.0, d = 0.429, P = 0.023), but no difference in TTO (d = 0.188, P = 0.778) or WTP (d = 0.299, P = 0.216). The folded F test showed significantly more variance among responses of cognitively impaired patients compared to unimpaired patients measured with the SG (0.39 vs. 0.21, F = 3.38 (10, 153), P = 0.001) and TTO (0.36 vs. 0.24, F = 2.26 (10, 153), P = 0.017). There was no difference in the preference variance of VAS (0.21 vs. 0.20, F = 1.08 (10, 153), P = 0.378) or WTP as a proportion of income (4.0 vs. 4.0, F = 1.00 (10, 153), P = 0.555).

There were marked differences in the correlation matrices of the preference measurement tools when stratified by cognitive status. In subjects without cognitive impairment, among the six possible pairings of preference measurement instruments, there were significant correlations between three pairings (VAS:TTO, rho = 0.19, P = 0.018; SG:TTO, rho = 0.36, P < 0.001; SG:WTP, rho = -0.33, P < 0.001) and a trend towards significance with another pairing (VAS:WTP, rho = 0.16, P = 0.054). In subjects with cognitive impairment, there was a significant correlation only between VAS and TTO scores (rho = 0.76, P = 0.023).

### Regression Models of Preferences

#### Visual Analogue Scale

Mean (SD) preferences for current health were 0.67 (0.20), i.e., on average, patients rated their current health equivalent to 67% of perfect health. There was a significant association between lower VAS scores and cognitive impairment (β = -0.12, P = 0.04, Table 2), but there was no association between VAS scores and patient characteristics or aneurysm history.

**Table 2:** Linear Regression Models of Patient Preferences

	**β Coefficients**				**Final Model**	
**Preference Measure**	Cognitive impairment	Prior aneurysm treatment	Income (2003 $US)	Constant	R^2^	F
**Visual Analogue Scale**	-0.12*	-	-	0.68***	0.02	0.048
**Standard Gamble**	-0.23**	-	-	0.79***	0.06	0.002
**Time Trade-Off**	-0.17*	0.08*	-	0.75***	0.04	0.028
**Willingness to Pay^†^**			2.02***	$34,000^†^	0.13	< 0.001

* P < 0.05

** P < 0.01

*** P < 0.001

^†^2003 $US

#### Standard Gamble

Mean (SD) preferences for current health were 0.78 (0.23), i.e., on average, patients were willing to accept up to a 22% risk of immediate death in return for a 78% chance of obtaining perfect health for the rest of their life. Multivariate regression modelling showed a significant independent association between lower SG values and cognitive impairment (β = -0.23, P = 0.002, Table 2). There was no association between SG values and patient characteristics or aneurysm history.

#### Time Trade-Off

Mean (SD) preferences for current health were 0.80 (0.25), i.e., on average, patients were willing to trade-off up to 4 years of expected survival to obtain 16 years of perfect health, followed by death. There was a significant independent association between lower TTO values and cognitive impairment (β = -0.17, P = 0.035), and an absence of previous aneurysm treatment (β = -0.08, P = 0.044; Table 2). There was no association between TTO values and patient characteristics or aneurysm history.

#### Willingness to Pay

Mean (SD) preferences for current health were $116,200 ($184,300), i.e., on average, patients were willing to pay up to 2.8 times their annual income to obtain perfect health. There was a significant association between higher WTP values (corresponding to lower health values) and greater income (β = 2.02, P < 0.001; Table 2). There was no association between WTP values and cognitive impairment, age, sex, race, education, or aneurysm history.

## Discussion

We measured preferences for current health using the VAS, SG, TTO, and WTP in a population of patients with cerebral aneurysms. We then looked at the association between preference values and cognitive functioning as assessed with the MMSE, patient characteristics, and aneurysm history. The MMSE classified 7% of our study population as cognitively impaired. The distributions of responses were different for unimpaired and cognitively impaired patients for the VAS, SG, and TTO. Cognitive impairment was associated with significant reduction in preferences for current health measured with the VAS, SG, and TTO. There was no association between cognitive impairment and difficulty in understanding or completing the preference measurement task.

There are several possible reasons that preference scores were lower in our patients with cognitive impairment. Patients with cognitive impairment may actually value their health state less because it includes a component of cognitive impairment. Alternatively, cognitive impairment may alter how patients respond to VAS, SG, and TTO and testing *per se*, biasing their responses downward independent of their "true" preferences. The two explanations are not mutually exclusive, and both could be operating in an additive or synergistic fashion. If our current measurement tools cannot accurately measure preferences in patients with cognitive impairment, then measuring the preferences of impaired individuals will require the development and validation of new instruments, and in the interim these individuals should be identified and excluded from preference analyses.

Cognitive impairment may well diminish preferences for current health – preferences vary with a variety of subject characteristics such as demographics [21,22], comorbid conditions [21,22], measurement instrument [23-25], mode of administration – computer versus personal interview [26], the population being tested – individuals with the condition of interest often provide higher values than others [27-29], and scale anchor points [30-32]. Neumann *et al. *used the Health Utilities Index Mark II to assess health values for Alzheimer's dementia from caregivers [10]. Health values were inversely related to patient health, ranging from 0.73 for questionable dementia to 0.14 for terminal dementia. Ekman and colleagues used the TTO and a postal survey to measure preferences for mild cognitive impairment and mild, moderate, and severe dementia health states in a cross section of the Swedish population [12]. Preferences varied inversely with cognitive functioning, ranging from 0.82 for mild cognitive impairment to 0.25 for severe dementia.

Jonsson and co-workers used the EuroQol 5D to measure preferences for current health in patients with Alzheimer's disease and proxy valuations from their primary caregivers [11]. Patient preferences varied little across MMSE-based severity levels, averaging 0.83. Proxy valuations were lower than patients' and varied inversely with the degree of dementia (range 0.69 for MMSE > 25 to 0.33 for MMSE < 10). In our regression models, cognitive impairment was associated with a 0.12 – 0.23 decrease in preference values, a substantial effect size. The consistent effect of cognitive impairment on preferences measured with three different techniques – SG, TTO, VAS – that differ widely in their cognitive demands provides cross-validating evidence in favour of a real detrimental effect of cognitive impairment on preferences for current health. We have no ready explanation why WTP preferences were not affected by cognitive impairment.

Cognitive impairment might interfere with comprehension and processing of information required to complete preference measurement tasks, leading to biased preference values. Woloshin and colleagues have shown that numeracy affects preferences measured with the SG, TTO, and VAS [33]. Bravata and colleagues showed that, even after excluding individuals with cognitive impairment based on the MMSE, the remaining subjects with relatively low MMSE scores were more likely to provide uniform preference values equal to 1.0 when asked to evaluate multiple hypothetical health states [13]. We found several differences between the patterns of responses of patients with cognitive impairment and those of unimpaired patients. The distributions of responses for our unimpaired subjects followed skewed-normal or skewed distributions with modal values at or near perfect health. In contrast, the preference distributions of our cognitively impaired subjects had non-standard morphologies and greater variance. This difference suggests that some cognitively impaired subjects may not have understood the test and given extreme or random responses (SG, TTO) or responses tending towards the middle of the visual scale (VAS). This pattern would result in lower mean preference scores compared to unimpaired patients, and may account for some of the differences between the two groups.

If there is a bias in preference reporting/measurement associated with cognitive impairment, one solution would be to exclude individuals with cognitive impairment from testing. Such a policy could be problematic for any assessments of societal preferences (which are recommended for use in cost-effectiveness analyses [23]), since it would exclude a substantial portion of the population – for example, an estimated 4.5 million people in the United States are afflicted with Alzheimer's disease [34]. The identification of cognitively impaired individuals would also be difficult. Adding a cognitive screening instrument to protocols collecting preference data would consume study resources and add to respondent burden. Our study used the MMSE, an 11-item instrument requiring 5–10 minutes and a face-to-face encounter. While widely used, the MMSE is not without its critics, and some authorities have suggested using a higher threshold to define cognitive impairment [35,36]. Other "bedside" alternatives to the MMSE are at least as complex and time consuming [37]. The 11-item Telephone Interview for Cognitive Status can be used for remote cognitive testing, but still requires 5–10 minutes to administer [38].

Twelve percent of our patient population had some difficulty understanding or completing the preference testing, although all provided responses for the VAS, SG, TTO, and WTP. Interestingly, we did not find that testing difficulties was associated with cognitive impairment as measured with the MMSE. Some investigators have excluded the responses of individuals who did not appear to understand the preference testing process [13,39,40], and others have developed techniques to detect and minimize inconsistencies during multiple preference measurements in the same subject [41]. Unfortunately, our study design did not provide us with sufficient data to allow a confident investigation of the effects of testing difficulties on preferences. Future investigations will include a more rigorous assessment of testing difficulties and enable investigation of the relationship between cognitive impairment and difficulty understanding and completing preference testing.

Most researchers have found that patient preferences vary depending on the measurement instrument, and our study is no exception – our patients had SG and TTO preferences significantly greater than VAS preferences (WTP values have a unique metric that precludes direct comparison with the other preference values).

These ubiquitous discrepancies have lead to a lively debate about their etiology and significance. Some believe that the SG is the "gold standard" in measuring patient preferences because it conforms to the axioms of von Neumann-Morgenstern utility theory; however, it is subject to bias and framing effects, and can be distorted by risk aversion [42-44]. The TTO has roots in decision theory and was developed as a more "user friendly" alternative to the SG, but TTO values can be confounded by time preferences [45-48]. While it is convenient to administer, the VAS has been criticized for lacking the theoretical underpinnings of the SG or TTO and may have limited applicability [49]. The VAS does not incorporate risk of death (SG) or certain reduced survival (TTO). Since most subjects are risk averse and somewhat reluctant to trade years of life, the VAS generally yields lower scores that the SG or TTO [50]. Finally, WTP responses are affected by economic resources, and WTP preferences are not expressed on a zero to one ratio scale, making it difficult to incorporate WTP values into decision analytic models [51,52]. Variations in risk aversion, time preferences, and economic resources are all likely contributing to the differences in preference values provided by the four instruments. We do not know whether one or more of these factors are asymmetrically distributed across our cognitively impaired and unimpaired patients, and it is unclear whether or how much these factors may be contributing to preference differences between cognitively impaired and unimpaired patients.

### Limitations

Our sample population was derived from patients with cerebral aneurysms under care at a single university hospital, and thus the results may not be generalizable to other patient populations. Logistical difficulties precluded the enrolment of all eligible patients into our study, and some who did enrol failed to complete all surveys. Relatively few of our patients were cognitively impaired, thus limiting our statistical power to determine the effects of cognitive impairment on preference measurements. Our patients exhibited only mild cognitive impairment: the mean MMSE score was 27.5, only 7% were cognitively impaired (MMSE score < 24), and only 1 patient had a MMSE < 20. In contrast, patients with Alzheimer's disease enrolled in studies have substantially lower mean MMSE scores (i.e., in the low 20's or high teens [53,54]); therefore our findings may not generalize to patients such as these with more severe cognitive deficits. Our data collection on subject difficulties with understanding or completing the preference instruments was sparse, limiting our analysis of testing difficulties.

## Conclusion

In our study population of patients with cerebral aneurysms, cognitive impairment was associated with lower preferences for current health when measured with three popular instruments – the standard gamble, time trade-off, and visual analogue scale. Further work is needed to assess whether lower preference values in these individuals represent a "real" decrement in preferences for a health state that includes a component of cognitive impairment or are the result of measurement bias related to cognitive deficits, or a combination of the two.

## Abbreviations

MMSE: Mini Mental State Examination; SAH: subarachnoid hemorrhage; SD: standard deviation; SG: standard gamble; TTO: time trade-off; VAS:visual analogue scale; WTP: willingness to pay.

## Competing interests

The authors declare that they have no competing interests.

## Authors' contributions

JTK was responsible for primary study design, supervision of data collection, primary data cleaning and analysis, manuscript drafting, and manuscript submission. JS served as a methodologic consultant, assisted with data analysis and interpretation, and participated in manuscript editing. MSR was a methodologic consultant, assisted with data analysis and interpretation, and participated in manuscript editing.
